# Serum Diamine Oxidase Values, Indicating Histamine Intolerance, Influence Lactose Tolerance Breath Test Results

**DOI:** 10.3390/nu14102026

**Published:** 2022-05-12

**Authors:** Wolfgang J. Schnedl, Nathalie Meier-Allard, Simon Michaelis, Sonja Lackner, Dietmar Enko, Harald Mangge, Sandra J. Holasek

**Affiliations:** 1 Practice for General Internal Medicine, Dr. Theodor Körnerstrasse 19b, 8600 Bruck an der Mur, Austria; 2 Division of Immunology and Pathophysiology, Otto Loewi Research Center, Medical University of Graz, Heinrichstrasse 31a, 8010 Graz, Austria; nathalie.allard@medunigraz.at (N.M.-A.); sonja.lackner@medunigraz.at (S.L.); sandra.holasek@medunigraz.at (S.J.H.); 3 Institute of Clinical Chemistry, Laboratory Medicine, Hospital Hochsteiermark, Vordernberger Straße 42, 8700 Leoben, Austria; simon.michaelis@kages.at (S.M.); enko.dietmar@gmx.at (D.E.); 4 Clinical Institute of Medical, Chemical Laboratory Diagnosis, Medical University of Graz, Auenbruggerplatz 30, 8036 Graz, Austria; harald.mangge@klinikum-graz.at

**Keywords:** irritable bowel syndrome, diamine oxidase, exhaled hydrogen, food intolerance, lactose intolerance

## Abstract

Lactose intolerance (LIT) is one of the major causes of irritable bowel syndrome (IBS) spectrum complaints. Differences in inadequate lactose digestion are described as various LIT phenotypes with basically unknown pathophysiology. In LIT patients, we retrospectively assessed the effect of histamine intolerance (HIT) on expiratory hydrogen (H_2_) during H_2_ lactose breath tests. In a retrospective evaluation of charts from 402 LIT patients, 200 patients were identified as having only LIT. The other 202 LIT patients were found to additionally have diamine oxidase (DAO) values of <10 U/mL, which indicates histamine intolerance (HIT). To identify HIT, standardized questionnaires, low serum DAO values and responses to a histamine-reduced diet were used. Patients were separated into three diagnostic groups according to the result of H_2_ breath tests: (1) LIT, with an H_2_ increase of >20 parts per million (ppm), but a blood glucose (BG) increase of >20 mg/dL, (2) LIT with an H_2_ increase of 20 ppm in combination with a BG increase of <20 mg/dL, and (3) LIT with an exhaled H_2_ increase of <20 ppm and BG increase of <20 mg/dL. Pairwise comparison with the Kruskal Wallis test was used to compare the areas under the curve (AUC) of LIT and LIT with HIT patients. Exhaled H_2_ values were significantly higher in H_2_ > 20 ppm and BG < 20 mg/dL patients with LIT and HIT (*p* = 0.007). This diagnostic group also showed a significant higher number of patients (*p* = 0.012) and a significant higher number of patients with gastrointestinal (GI) symptoms during H_2_ breath tests (*p* < 0.001). Therefore, low serum DAO values, indicating HIT, influence results of lactose tolerance breath tests.

## 1. Introduction

Lactose intolerance (LIT) is the inability to properly digest the disaccharide lactose, which is mainly found in milk and dairy products [1]. Depending on the ingested quantity of lactose and the activity of the gastrointestinal (GI) mucosal enzyme lactase, patients with LIT may experience symptoms on the irritable bowel syndrome (IBS) spectrum [2]. However, if not-absorbed, lactose reaches the large intestine and it is used as a bacterial substrate, causing fermentation through microbiota. Approximately half of LIT patients with GI complaints of the IBS spectrum require not only reduced lactose intake but should also follow some additional diet modifications to improve GI complaints [3]. In HIT, a disproportionate amount of histamine in the body is thought to result from the consumption of food with high histamine content, and a mainly reduced ability of the GI enzyme DAO that seems responsible for the digestion of histamine [4]. The clinical diagnosis of HIT is challenging, and serum DAO values are believed to not adequately reflect gastrointestinal DAO activity. Nonetheless, HIT diagnosis may be supported with the measurement of the enzyme DAO in serum [5]. However, an association of DAO and lactase activities in small intestinal biopsies of children was shown [6]. We observed in patients with high end-expiratory H_2_ levels with LIT an additional food intolerance/malabsorption compared to LIT-only [5]. Subsequently, we have retrospectively evaluated 402-LIT patients with H_2_ breath tests, and included the serum DAO measurements to find additional HIT. Comparing ‘LIT only’ to ‘LIT with HIT’ patients, we searched for differences in exhaled H_2_ and GI symptoms during H_2_ breath tests, and differences in numbers of patients. Thus, an influence of low serum DAO values on results of lactose tolerance breath tests has been demonstrated.

## 2. Materials and Methods 

In a retrospective study of charts from LIT patients, we identified 402 consecutive patients who were evaluated for extra HIT. In our outpatient facility, all here included patients presented with nonspecific, non-allergic functional gastrointestinal (GI) complaints of the IBS spectrum. GI symptoms indicated were abdominal pain, flatulence, loose stools, diarrhea, and postprandial fullness. The patients had not previously had a diet. The timely relation to the ingestion of food, including pharmaceutical treatments that might influence symptoms of the IBS spectrum was evaluated.

Lactose H_2_ breath tests were completed with the Gastrolyzer (Bedfont Scientific Inc., Kent, UK) using 200 mL water with 50 g of lactose. End-expiratory exhalation of H_2_ was determined every 30 min (minutes) for a period of 120 min after fasting. Blood glucose (BG) was measured every 60 min for a period of 2 h. A detailed anamnesis, a standardized questionnaire concerning complaints of HIT and a response to a histamine-reduced diet was used. To identify food intolerance/malabsorption or combinations thereof, we include serum DAO measurements in the assessment of patients presenting with recurring complaints of the IBS spectrum. DAO in serum was determined with the radio extraction assay DAO Rea 100 (Sciotec Diagnostic Technologies, Tulln, Austria).

No antibiotics or laxatives were allowed before the breath tests. No colonoscopy was performed within the prior four weeks. At the first presentation, blood drawings were done after overnight fasting (>12 h). The H_2_ lactose breath test was started without having smoked, chewed gum or performed vigorous exercise overnight. 

Additionally, all included patients were tested for fructose malabsorption (FM), *Helicobacter pylori* infection and celiac disease. FM was searched with H_2_ breath tests using a drink containing 25 g of fructose. Histologic evaluation of the gastric mucosa and/or an enzyme-linked IgA immunosorbent assay (ELISA, Serion, Würzburg, Germany) were performed to detect *Helicobacter pylori* infection. For the presence of celiac disease, antibodies against tissue transglutaminase were measured with the anti-tTG IgA ELISA (Euro Diagnostica AB, Malmö, Sweden). Patients with alarming symptoms such as unintentional weight loss, rectal bleeding, or recent changes in bowel function were not included in this evaluation. At the time of diagnosis, written information on LIT and, if present, on HIT was handed to the patients. Additionally, a registered dietician was asked to start a food intolerance/malabsorption-reduction and/or -elimination diet to the symptomatology. For long-term symptom reduction, the dietician observed the individual diet. 

The Ethical Committee of the Johannes Kepler University in Linz, Austria, approved the study (No. K-107-16). The study follows the ethical guidelines of the Declaration of Helsinki.

## 3. Statistical Analysis

The data distribution was assessed with the Shapiro Wilk test. Not normally distributed data were presented as medians and interquartile ranges (IQR). The areas under the curve (AUC) of exhaled hydrogen during H_2_ breath tests were calculated and compared with a Mann-Whitney U test. The Chi-square and the Fisher’s exact test were applied for the evaluation of symptoms. The level of significance was set to 5%. Bonferroni correction was used for multiple testing.

Statistical analyses were performed with IBM SPSS statistics version 26.0 (IBM, Armonk, NY, USA), and GraphPad Prism version 9.0.0. (GraphPad Software, San Diego, CA, USA) was used to generate figures.

## 4. Results

During this retrospective evaluation of 402 lactose-intolerant patients, a 155/247 male/female ratio, a median age of 43 years, and age range 17–86 years were found. We studied 200 patients—male/female 83/117, median age 49 years, age range 17–86 years—with LIT only. Their DAO values were >10 U/mL (median 19.4 U/mL, range 10.1–80). We observed a striking number of patients with high end-expiratory H_2_ levels with LIT and HIT, especially in the diagnostic group characterized by >H_2_ of 20 ppm and a BG < 20 mg/dL. Therefore, patients were divided into three diagnostic groups according to the results of H_2_ breath tests: (1) LIT with an H_2_ increase of >20 ppm, but a BG increase of >20 mg/dL; (2) LIT with an H_2_ increase of >20 ppm in combination with a BG increase of <20 mg/dL; and (3) LIT with an exhaled H_2_ increase of <20 ppm and BG increase of <20 mg/dL.

All values of the lactose tolerance tests for exhaled H_2_ and measured BG are shown in Table 1 and Table 2, respectively. In the category LIT-only we found 29 patients with increasing expiratory H_2_ values from the baseline >20 ppm and their BG values increased >20 mg/dL from fasting BG prior to the test (male/female 13/16, median age 57 years, age range 23–86 years). With an increase >20 ppm of expiratory H_2_ and BG increase of <20 mg/dL, 85 patients were included in the study (male/female 33/52, median age 51 years, age range 17–86 years). With BG increase <20 mg/dL, 86 patients were found (male/female 37/49, median age 43 years, age range 17–83 years). During the test, their expiratory H_2_ values increased <20 ppm. 

However, in 202-LIT patients, additional values for DAO in serum were <10 U/mL (median 6.1 U/mL, range 0.2–9.9), suggestive for HIT (male/female 72/130, median age 41 years, age range 18–82 years). In 27 patients we found LIT and HIT diagnosed with only increasing expiratory H_2_ values from the baseline > 20 ppm (male/female 8/19, median age 41 years, age range 21–82 years). Their BG values increased >20 mg/dL from prior fasting BG. With an increase >20 ppm of expiratory H_2_ from the baseline and BG increase of <20 mg/dL, 63 patients were included in the study (male/female 25/38, median age 43 years, age range 18–82 years). With BG < 20 mg/dL increase 112 patients were found (male/female 39/73, median age 39 years, age range 20–77 years). Their expiratory H_2_ values increased from the baseline < 20 ppm. 

Using the Mann-Whitney U test, the AUCs demonstrated no significant difference comparing all LIT and LIT with HIT patients (*p* = 0.36). The analysis of the AUCs with an increase >20 ppm of expiratory H_2_ and BG increase of <20 mg/dL revealed that the AUC was significantly higher in patients diagnosed with LIT and HIT compared to LIT only patients (*p* = 0.007). 

**Figure 1:**
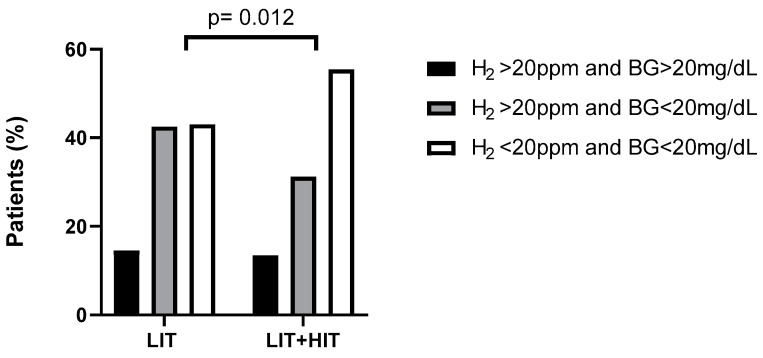
Numbers of patients in percent (%). Included are 402 patients, consisting of 200 LIT-only patients and 202-LIT patients with additional HIT. Abbreviations: BG, blood glucose; H_2_, hydrogen; HIT, histamine intolerance; LIT, lactose intolerance; ppm, parts per million.

Numbers of patients in percent according to diagnostic groups are shown in Figure 1. A significant difference was found comparing LIT-only to LIT and HIT in the diagnostic groups H_2_ > 20 ppm and BG < 20 mg/dL and H_2_ < 20 ppm and BG < 20 mg/dL (*p* = 0.012).

Patients indicated their symptoms during the breath tests on paper. GI symptoms included bloating, abdominal pain, stomach rumbling, diarrhea, fullness, belching and heartburn. Extra-intestinal symptoms in LIT-only patients included headache, nausea, vertigo, fatigue, hunger and mucus in the throat. LIT and HIT patients showed extra-intestinal symptoms as headache, nausea, vertigo, fatigue, a swollen eyelid, eczema, palpitation and dyspnea. 

**Figure 2:**
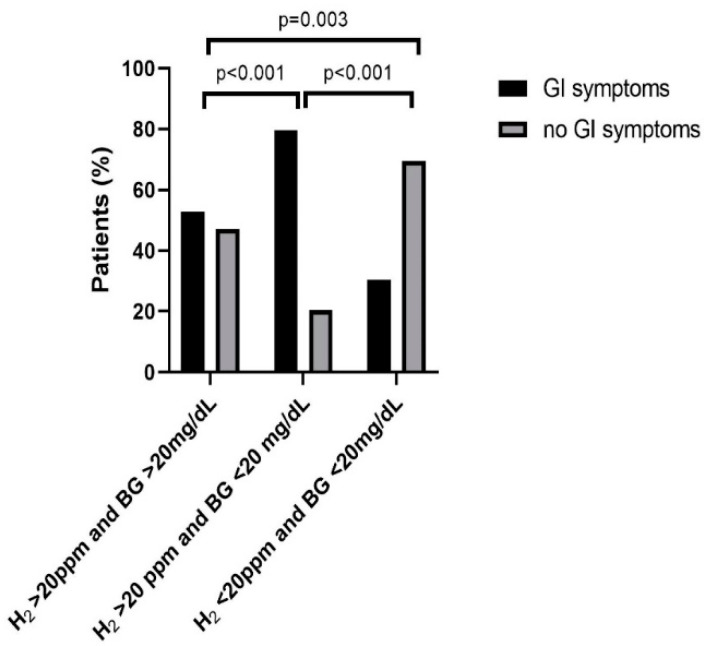
Three hundred eighty five of 402-LIT patients indicated GI symptoms during their lactose H_2_ breath tests. Abbreviations: BG, blood glucose; GI, gastrointestinal; H_2_, hydrogen; LIT, lactose intolerance; ppm, parts per million.

As shown in Figure 2, 28 out of the 53 patients (53%) with H_2_ > 20 ppm and BG > 20 mg/dL, 113 of 142 (80%) with H_2_ > 20 ppm and BG < 20 mg/dL, and 58 out of 90 (64%) with H_2_ < 20 ppm and < 20 mg/dL indicated GI symptoms during lactose breath tests. The diagnostic groups’ pairwise comparison showed a significantly higher number of patients with GI symptoms during H_2_ breath tests comparing H_2_ > 20 ppm and BG < 20 mg/dL with H_2_ > 20 ppm and BG > 20 mg/dL (*p* < 0.001; *n* = 195). Patients with H_2_ < 20 ppm and BG < 20 mg/dL had less GI symptoms compared to >20 ppm and BG > 20 mg/dL (*p* = 0.003; *n* = 243) and to H_2_ > 20 ppm and BG < 20 mg/dL (*p* < 0.001; *n* = 332). 

Neither FM nor *Helicobacter pylori* infection were found in included patients. Antibodies against tissue transglutaminase were not present. All patients that were >50 years of age were examined by colonoscopy and no pathology was present.

## 5. Discussion

Currently, IBS is classified according to the Rome IV criteria as a functional GI disorder, but its pathophysiology remains unknown and various disease models are continuously being proposed [7]. IBS symptoms are associated with a high symptom burden and impaired quality of life [8]. Furthermore, IBS symptoms may resemble food intolerance/malabsorption complaints [2], and lactose is among the known triggers causing IBS symptoms [9]. However, IBS patients also believe that foods, especially histamine in foods, cause their GI symptoms [10]. Thus, it is assumed that LIT may be combined, as part of a wider intolerance, with variably absorbed and/or digested additional food ingredients. This appears in more than half of patients with complaints on the IBS spectrum and requires not only the restriction of lactose intake but also additional diets to improve GI complaints [3]. Various combinations of food malabsorption/intolerance [5], including infection with *Helicobacter pylori* [11], were demonstrated to influence LIT breath test results in patients with IBS spectrum complaints. Here we demonstrate that, in our evaluation, at least 50 percent of consecutive LIT patients have additional food intolerance/malabsorption with low serum diamine oxidase (DAO) values, indicating additional histamine intolerance (HIT).

LIT is present in most countries, although locally varying it has an estimated global prevalence of up to 70 percent [8]. Persistence of the lactose-degrading enzyme lactase is associated with the beginning of agriculture in Europe. Populations that domesticated dairy animals and consumed their milk had a higher frequency of mutations associated with the expression of lactase. Maintaining intestinal lactase into adulthood is combined with the expression of polymorphisms in the gene encoding lactase located on chromosome 2q21. The presence of this enzyme enables digestion by degrading the disaccharide lactose into its monosaccharides, glucose and galactose. Lactase persistence is inherited as a dominant Mendelian trait and, apparently, the consumption of dairy products meant a nutritional advantage in ancient times [12]. The occurrence of continuing adult lactase activity was estimated to be 7500–10,000 years ago. The Tyrolean Iceman, named Ötzi, a 5300-year-old Copper age male, was probably lactose intolerant, as a polymorphism responsible for lactase persistence was found in his genome [13]. However, the genetic determination of polymorphisms for lactase persistence does not allow the assumption that digestive complaints caused by ingested lactose are present to be drawn. Symptoms in LIT may appear when the sugar passes through the intestines without being degraded [8]. The lactose then reaches the microbiota, where it serves as a bacterial substrate. This leads to fermentation with hydrogen production. Therefore, the clinical diagnosis of LIT is mainly performed with lactose using hydrogen breath tests [14].

Although the interest in HIT is increasing, further investigations are certainly needed [4,15]. An impaired degradation of ingested histamine due to an anticipated GI mucosal DAO deficiency causes a disturbed gut flora [16]. We use serum DAO measurements besides using a thorough anamnesis, a standardized questionnaire and a response to histamine-reduced diet for the diagnosis of HIT [13]. Nevertheless, serum DAO values are not believed to reflect gastrointestinal DAO activity [17]. Nonetheless, increasing evidence of a relationship of serum DAO to GI activity has been described [18,19,20], and it has been demonstrated that a strict histamine-reduced diet may increase serum DAO values [21]. Although improvements of determination methods for serum DAO are also necessary [22] additional indications for an association of serum DAO to digestion are shown here.

Differences in adequate lactose digestion are recognized. Because these phenotypic differences are with unknown pathophysiology, various terms are being used to describe different LIT phenotypes. Included are lactase persistence, lactase non-persistence, lactase deficiency, lactose maldigestion, lactose sensibility and lactose intolerance [23]. Generally, patients demonstrating GI complaints caused by ingestion of lactose are being termed lactose intolerant. However, in LIT the relationship to IBS symptoms is still unclear [24]. In adults, the varying ability to digest lactose seems to divide people into various phenotypes [25]. 

However, symptoms depend only on the patients’ subjective interpretation. Subjective perception of food intolerance/malabsorption does not always mean LIT [24]. Since there are known differences in the clinical appearance of LIT, some phenotypes may be caused by a varying digestive response between isolated lactose and whole milk. LIT-caused symptoms seem influenced by the food matrix of milk or by individual GI digestion and absorption properties [26]. Milk has been used as a substrate to find LIT in several studies, and the additional milk ingredients besides lactose, including fats, may reduce gastric emptying and influence LIT. Recent studies have suspected that milk protein β-casein variants, A1 and A2 β-casein, affect LIT [27]. In general, we detected low serum DAO levels with high end-expiratory H2 levels, and this was also described as an LIT phenotype [28]. 

Treatment of LIT includes the reduction of the dietary amount of lactose with a lactose-reduced or lactose-free diet until symptoms are absent. Nonetheless, histamine may accumulate in dairy products as a result of lactic acid bacteria and yeast metabolism that contributes to the ripening and flavoring [29]. Subsequently, in patients with LIT and not-diagnosed HIT who are using a lactose-free diet, ripened dairy products may cause HIT-related GI symptoms [30]. So far, therapeutic measures for HIT include a histamine-reduced diet and the use of oral DAO to relieve symptoms in patients with HIT [19]. Overall, endoscopy with biopsies and histologic evaluation of the gastric and intestinal mucosa, radiology, and ultrasound are non-interchangeable methods for ruling out organic disease, especially in patients older than 50 years. However, we present a single center experience, and a selection bias cannot be excluded.

We observed that LIT patients with serum DAO values < 10 U/mL had high end-expiratory H_2_ during lactose breath tests. In addition, an association between DAO and lactase activities in small intestinal biopsies from children was demonstrated several years ago [6]. Here we demonstrate detailed evidence that low serum DAO levels, indicative of HIT, affect lactose breath test results. In LIT with HIT patients, the diagnostic group with H_2_ > 20 ppm and BG < 20 mg/dL showed the highest increase in exhaled H_2_ (Figure 1), a significant higher number of patients (Table 1) and a significant higher number of patients with GI symptoms, during H_2_ breath tests (Figure 2). This suggests not only differences in gastrointestinal mucosa, but also differences in metabolism based on different abilities to digest and/or absorb lactose and histamine in the gut. However, the role of HIT combined with LIT and their interaction remains to be further evaluated. 

High end-expiratory H_2_ levels on lactose breath tests increase the likelihood of additional food intolerance/malabsorption and/or also *Helicobacter pylori* infection [5,11]. We suggest that in LIT patients, HIT offers an additional option for patients to put complaints of the IBS spectrum into context. When establishing dietary restrictions for each LIT and HIT patient, individual tolerance to the single or the combination of food intolerance/malabsorption needs consideration. Subsequently, a registered and experienced dietitian is essential in developing an individualized diet tailored to minimize the symptoms and achieve long-term symptom reduction.

## Figures and Tables

**Table 1:** Median blood glucose values (mg/dL) during lactose H_2_ breath tests in 402 LIT patients.

LIT	*n*	Fasting BG mg/dL Median (IQR) Min-Max	60 min. BG mg/dL Median (IQR) Min-Max	120 min. BG mg/dL Median (IQR) Min-Max
H_2_ > 20 ppm + BG increase > 20 mg/dL	29	95 (23) 75–131	127 (28) 95–182	105 (19) 70–170
H_2_ > 20 ppm + BG increase < 20 mg/dL	85	99 (15) 76–149	107 (19) 82–158	102 (17) 69–156
H_2_ < 20 ppm + BG increase < 20 mg/dL	86	103 (17) 77–140	106 (18) 79–137	98 (16) 72–129
**LIT + HIT**				
H_2_ > 20 ppm + BG increase > 20 mg/dL	27	92 (22) 77–162	127 (16) 97–196	107 (23) 75–173
H_2_ > 20 ppm + BG increase < 20 mg/dL	63	100 (15) 73–122	104 (18) 83–131	98 (13) 76–125
H_2_ < 20 ppm + BG increase < 20 mg/dL	112	99 (14) 74–134	105 (12) 58–136	96 (13) 63–120

Abbreviations: BG, blood glucose; H_2_, hydrogen; HIT, histamine intolerance; IQR, interquartile range (Q1–Q3); LIT, lactose intolerance; min., minutes; *n*, number of patients; ppm, parts per million.

**Table 2:** Median parts per million (ppm) of exhaled H_2_ during lactose breath tests in 402 LIT patients.

LIT	*n*	Fasting ppm Median (IQR) Min-Max	30 min. ppm Median (IQR) Min-Max	60 min. ppm Median (IQR) Min-Max	90 min. ppm Median (IQR) Min-Max	120 min. ppm Median (IQR) Min-Max
H_2_ > 20 ppm + BG increase > 20 mg/dL	29	3 (6) 0–13	5 (5) 0–25	17 (23) 1–73	32 (39) 6–316	46 (35) 26–190
H_2_ > 20 ppm + BG increase < 20 mg/dL	85	5 (9) 0–23	9 (13) 0–163	20 (39) 0–156	52 (56) 0–179	64 (62) 0–243
H_2_ < 20 ppm + BG increase < 20 mg/dL	86	4 (6) 0–17	4 (6) 0–16	3 (5) 0–15	3 (4) 0–21	4 (4) 0–23
**LIT + HIT**						
H_2_ > 20 ppm + BG increase > 20 mg/dL	27	4 (7) 0–16	8 (9) 0–147	26 (40) 1–74	53 (85) 5–136	59 (69) 10–188
H_2_ > 20 ppm + BG increase < 20 mg/dL	63	3 (7) 0–20	7 (15) 0–56	34 (48) 0–208	74 (87) 12–265	89 (91) 16–361
H_2_ < 20 ppm + BG increase < 20 mg/dL	112	4 (6) 0–24	4 (6) 0–22	3 (5) 0–33	4 (7) 0–33	4 (7) 0–33

Abbreviations: BG, blood glucose; H_2_, hydrogen; HIT, histamine intolerance; IQR, interquartile range (Q1–Q3); LIT, lactose intolerance; min, minutes; *n*, number of patients; ppm, parts per million.

## Data Availability

The data that support the findings of this study are available from the corresponding author upon reasonable request.
